# Long-Term Oncological Outcomes After Colorectal Anastomotic Leakage: A Retrospective Dutch Population-based Study

**DOI:** 10.1097/SLA.0000000000005647

**Published:** 2022-08-05

**Authors:** Melissa N.N. Arron, Nynke G. Greijdanus, Sarah Bastiaans, Pauline A.J. Vissers, Rob H.A. Verhoeven, Richard P.G. ten Broek, Henk M.W. Verheul, Pieter J. Tanis, Harry van Goor, Johannes H.W. de Wilt

**Affiliations:** * Department of Surgery, Radboud University Medical Centre, Radboud Institute for Health Sciences, Nijmegen, the Netherlands; † Department of Research and Development, Netherlands Comprehensive Cancer Organization (IKNL), Utrecht, the Netherlands; ‡ Department of Medical Oncology, Amsterdam UMC, Cancer Centre Amsterdam, University of Amsterdam, Amsterdam, the Netherlands; § Department of Medical Oncology, Radboud Institute for Health Sciences, Radboud University Medical Centre, Nijmegen, the Netherlands; ∥ Department of Surgery, Amsterdam UMC, Cancer Centre Amsterdam, University of Amsterdam, Amsterdam, the Netherlands; ¶ Department of Surgical Oncology and Gastrointestinal Surgery, Erasmus Medical Centre, Rotterdam, the Netherlands

**Keywords:** anastomotic leakage, colorectal cancer, disease-free survival, disease recurrence, oncological outcomes, relative survival

## Abstract

The aim was to evaluate the impact of anastomotic leak (AL) after colon cancer (CC) and rectal cancer (RC) surgery on 5-year relative survival, disease-free survival (DFS), and disease recurrence.

**Background:**

AL after CC and RC resection is a severe postoperative complication with conflicting evidence whether it deteriorates long-term outcomes.

**Methods:**

Patients with stage I to IV CC and RC who underwent resection with primary anastomosis were included from the Netherlands Cancer Registry (2008–2018). Relative survival, measured from day of resection, and multivariable relative excess risks (RERs) were analyzed. DFS and recurrence were evaluated in a subset with stage I to III patients operated in 2015. All analyses were performed with patients who survived 90 days postoperatively.

**Results:**

A total of 65,299 CC and 22,855 RC patients were included. Five-year relative survival after CC resection with and without AL was 95% versus 100%, 89% versus 94%, 66% versus 76%, and 28% versus 25% for stage I to IV disease. AL was associated with a significantly higher RER for death in stage II and III CC patients. Stage-specific 5-year relative survival in RC patients with and without AL was 97% versus 101%, 90% versus 95%, 74% versus 83%, and 32% versus 41%. AL was associated with a significantly higher RER for death in stage III and IV RC patients. DFS was significantly lower in CC patients with AL, but disease recurrence was not associated with AL after colorectal cancer resection.

**Conclusion:**

AL has a stage-dependent negative impact on survival in both CC and RC, but no independent association with disease recurrence.

Anastomotic leakage (AL) is the most feared complication following colorectal cancer (CRC) resection. The incidence of AL varies between 3% and 20%, depending on the type of resection, anastomosis location, neoadjuvant treatment, and sex.^1^–^5^ AL is associated with severe short-term morbidity,^6^,^7^ and ~12% of colon cancer (CC) patients and 2% of rectal cancer (RC) patients die within the postoperative period after developing AL.^8^

Although it is widely recognized that AL is associated with poor short-term outcomes, the association with long-term (oncological) outcomes is ambiguous.^9^–^11^ Several studies demonstrated an association between AL after CRC resection and decreased survival, with long-term cancer-specific mortality hazard ratios (HR) up to 1.75,^3^,^12^,^13^ but others failed to confirm this association.^10^,^14^ In addition, while CC and RC are considered as separate entities most of the previous studies did not discriminate between them. Moreover, the majority of studies used overall survival, but relative survival gives a better representation of the approximation of cancer-specific survival because it adjusts for the general life expectancy. Consequently, the literature should be interpreted with caution.

Evidence is also inconclusive regarding the impact of AL on disease-free survival (DFS) and disease recurrence.^15^–^17^ The Colorectal cancer laparoscopic or open resection I and II trials demonstrated no association between AL and disease recurrence in CC patients. Contrary, in RC patients AL was associated with decreased DFS and increased local recurrence rates.^15^ Their relatively small study populations makes it difficult to extrapolate these conclusions.

Investigating the impact of AL on long-term oncological outcomes can provide an important basis for future studies to investigate diagnosis strategy and treatment strategy. This nationwide study aimed to evaluate the impact of AL on 5-year relative survival, DFS, and disease recurrence after restorative CC- and RC resection.

## METHODS

This population-based observational study included CRC patients diagnosed between January 1, 2008 and December 31, 2018 from the Netherlands Cancer Registry (NCR), which is maintained by the Netherlands Comprehensive Cancer Organization (IKNL). The following patient, tumor, and treatment characteristics are extracted from medical files: sex, age, American Society of Anesthesiologists (ASA) classification, body mass index, tumor location, pathological tumor stage, (neo)adjuvant therapy, type of surgical resection, and surgical approach. Registered postoperative outcomes consisted of AL, readmission <60 days and mortality. Follow-up regarding vital status was completed on January 31, 2020 and was captured by linking of the NCR to the Municipal Personal Records Database. Additional patient record review was performed to collect data on disease recurrence for patients diagnosed with stage I to III CRC between January 1, 2015 and June 30, 2015. Approval was obtained by the scientific board of the Prospective National Colorectal Cancer Cohort and the privacy review board of IKNL. Ethical approval and informed consent was not required according to the Dutch law.

### Inclusion and Exclusion Criteria

Patients with CRC stage I to IV who underwent surgical resection with formation of a primary anastomosis were included. Patients were excluded if no primary anastomosis was created (transanal endoscopic microsurgery, abdominoperineal resection, and Hartmann procedure). Patients who died within 90 days after surgery were also excluded from analyses, to prevent bias from death due to surgical complications (Supplementary Fig. 1, Supplemental Digital Content 2, http://links.lww.com/SLA/E116).

### Definitions

AL was defined as leakage of abdominal content or abscess formation at the anastomosis requiring reoperation, radiological intervention or readmission within 60 days after surgery. This definition encompasses grade B to C leakages according to the ISREC classification.^18^ Surgical procedures for CC included ileocecal resection, right or left hemicolectomy, transversectomy, sigmoid resection, or subtotal colectomy. RC resections comprised (low) anterior resection and partial mesorectal excision. Staging of the primary tumor was done using the UICC TNM classification according to the 6th (2008/2010), 7th (2010/2017), and 8th edition (2017/2018). The International Classification of Disease-Oncology was used to classify anatomical location of the primary tumor and metastases. Tumors were classified based on cancer cell differentiation into: well differentiated, moderately differentiated, poor differentiated, and anaplastic.

### Outcomes

The primary outcome was 5-year relative survival, measured from day of surgical resection. Relative survival was defined as the ratio of the proportion of CRC survivors to the proportion of expected CRC-free survivors in the general Dutch population based on same sex, age, and calendar year. Secondary outcomes were DFS and disease recurrence. DFS was defined as time from diagnosis to recurrent disease or death within 4 years after primary surgery. Disease recurrence encompasses: local, distant, or local with distant recurrence. Recurrent disease was diagnosed with imaging or at reoperation, and confirmed by histopathology.

### Statistical Analysis

Separate analyses were performed for CC and RC patients and compared between patients with and without AL. Descriptive statistics were used to report patient and tumor characteristics. Categorical data was presented as frequencies with percentages and continuous data was presented as mean with SD or median with interquartile range (IQR), depending on the distribution. χ^2^ and independent *t* tests were used to assess differences in characteristics between patients with and without AL. Relative survival was calculated using the Ederer II method.^19^,^20^ Differences in relative survival between patients with and without AL were assessed with a 2-sample proportion test. Multivariable relative excess risks (RERs) were estimated with 95% confidence intervals (CI) to determine the association between AL and excess risk of death. RERs for death were adjusted for sex, age (<70 and ≥70 y), surgical approach, tumor stage, type of resection, neoadjuvant (chemo)radiation (RC) and adjuvant chemotherapy (CC). DFS survival and disease recurrence were analyzed in a subset cohort of patients diagnosed with stage I to III CRC in the first semester of 2015. The association between AL and DFS was presented in Kaplan-Meier curves with log-rank test. Disease recurrence and death were counted as an event. Patients alive at the end of the study or loss to follow-up were censored. Univariable and multivariable cox proportional hazard regression analysis were performed to assess the association with disease recurrence. Confounders that were significantly associated with disease recurrence in the univariable analysis or with clinical relevance (ie, AL) were included in the multivariable analysis (presented with HR and 95% CI). Statistical significance was defined as a 2-sided *P*-value of <0.05. Relative survival and RER calculation was performed in Stata version 16.0, StataCorp LLC, College Station, TX, IBM SPSS Statistics version 25.0, IBM Corp, Armonk, NY.

## RESULTS

### Baseline Characteristics

Baseline characteristics are presented in Table 1. A total of 100,383 patients underwent a CRC resection of whom 92,304 patients underwent CRC surgery with formation of a primary anastomosis between 2008 and 2018, comprising 68,891 CC patients and 23,413 RC patients. In the CC cohort 3,552 patients died within 90 days postoperatively, of whom 723 with AL (20.4%) and 2829 (79.6%) without AL and survival data of 40 patients was missing, resulting into a total of 65,299 CC patients included in this study (3136 patients with AL and 62,163 without AL). In the RC cohort 546 patients died within 90 days postoperatively, including 127 with AL (23.3%) and 419 without AL (76.7%) and survival data of 12 patients was missing, resulting into a total of 22,855 RC patients included in this study (1814 with AL and 21,041 without AL).

**TABLE 1:** Baseline Patient, Tumor and Treatment Characteristics of the Included Patients (Excluding Patients That Died Within 90 d After Surgery)

	Colon Cancer Patients			Rectum Cancer Patients		
	With AL (N=3136)	Without AL (N=62,163)	*P*	With AL (N=1814)	Without AL (N=21,041)	*P*
Male sex	1906 (5.7)	31,570 (94.3)	**<0.01**	1431 (9.5)	12,747 (90.5)	**<0.01**
Age <70 y	1580 (5.2)	29,209 (94.5)	**<0.01**	1268 (8.9)	12,917 (91.1)	**<0.01**
Setting						
Elective	2934 (4.8)	58,348 (95.2)	0.50	1808 (8.0)	20,893 (92.0)	0.07
Urgent/emergency	202 (5.0)	3815 (95.0)		6 (3.9)	148 (96.1)	
Surgical approach						
Open	1305 (5.3)	23,475 (94.7)	**0.01**	506 (7.6)	6133 (92.4)	0.12
Laparoscopic	1507 (4.6)	31,188 (95.4)		1091 (8.3)	11,979 (91.7)	
Robot-assisted	3 (1.3)	225 (98.7)		27 (9.9)	246 (90.1)	
Unknown	380	7513		199	2725	
Pathological tumor stage						
Stage 1	595 (4.2)	13,445 (95.8)	**<0.01**	409 (7.7)	4872 (92.3)	0.44
Stage 2	1190 (5.1)	22,262 (94.9)		452 (7.9)	5292 (92.1)	
Stage 3	980 (4.8)	19,255 (95.2)		822 (8.2)	9172 (91.8)	
Stage 4	363 (4.9)	6988 (95.1)		127 (7.2)	1634 (92.8)	
Missing	9	222		4	74	
Tumor differentiation						
Well differentiated	125 (4.6)	2574 (95.4)	0.76	49 (7.1)	640 (92.9)	0.26
Moderately differentiated	2285 (4.8)	45,190 (95.2)		1220 (8.1)	13,904 (91.9)	
Poor differentiated/anaplastic	416 (4.7)	8522 (95.3)		115 (9.1)	1147 (90.9)	
Missing	384	6191		463	5465	
Type of resection						
Ileocecal resection/right hemicolectomy	1666 (4.2)	37,595 (95.8)	**<0.01**			
Transversectomy	103 (7.0)	1373 (93.0)				
(Extended) left hemicolectomy	169 (4.9)	3293 (95.1)				
Sigmoid resection	1036 (5.3)	18,410 (94.7)				
Subtotal resection	162 (9.8)	1492 (90.2)				
(Low) anterior resection/partial mesorectal excision				1795 (7.9)	20,795 (92.1)	—
Neoadjuvant radiotherapy						
No				761 (6.9)	10,337 (93.1)	**<0.01**
Yes				1053 (9.0)	10,704 (91.0)	
Neoadjuvant chemoradiation						
No				1439 (7.9)	16,742 (92.1)	0.81
Yes				375 (8.0)	4299 (92.0)	
Adjuvant chemotherapy*						
No	500 (7.4)	6274 (92.6)	**<0.01**			
Yes	466 (3.5)	12,801 (96.5)				

Bold values indicate a significance level of *P*<0.05.

* Only patients with stage III colon cancer.

The total incidence of AL after CC resection was 5.6% (3859/68,891). After excluding patients who died within 90 days postoperatively it was 4.8% (3136/65,299). Male sex and age below 70 years were associated with a higher AL rate after CC resection (*P*<0.01). Incidence of AL was significantly different between pathological tumor stages (I–IV), surgical approaches and types of resection. In the stage III CC group, 48% (466/966) of the patients with AL received adjuvant chemotherapy compared with 67% (n=12,801/19,075) of the patients without AL (*P*<0.01). The total incidence of AL after RC resection was 8.3% (1941/23,413). After excluding patients who died within 90 days postoperatively it was 7.9% (n=1814/22,855). Male sex, age below 70 years and neoadjuvant radiotherapy were associated with AL after RC resection (*P*<0.01).

### Relative Survival

**FIGURE 1:**
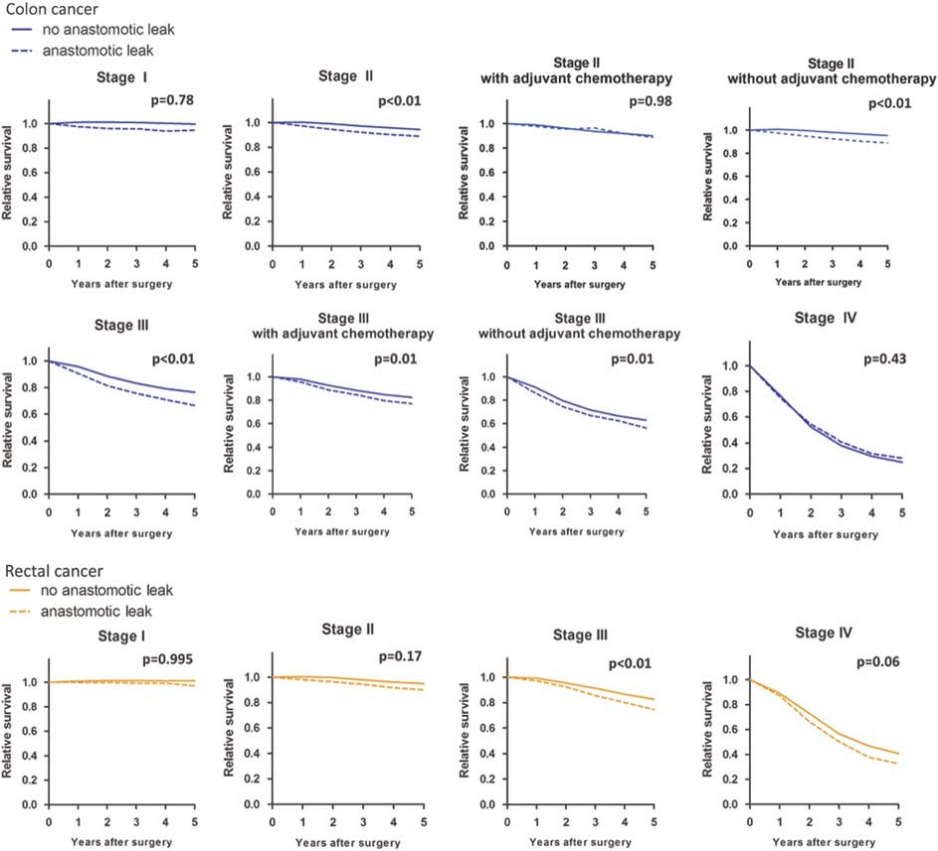
Five-year relative survival after CC and RC resection calculated using Ederer II method. Dotted line indicates patients with AL, continuous line indicates patients without AL. Blue lines: colon cancer patients, Orange lines: rectal cancer patients.

Relative 5-year survival for CC patients with or without AL was 95% versus 100% for stage I (HR: 1.37, 95% CI: 0.16–12.13, *P*=0.78), 89% versus 94% for stage II (HR: 1.61, 95% CI: 1.12–2.32, *P*=0.01), 66% versus 76% for stage III (HR: 1.55, 95% CI: 1.34–1.78, *P*<0.01), and 28% versus 25% for stage IV (HR: 0.95, 95% CI: 0.83–1.08, *P*=0.43, Fig. 1). Multivariable RER for death after CC resection was significantly higher for patients with AL (Table 2). Stage II CC patients with AL who were not treated with adjuvant chemotherapy had a higher RER for death compared with CC patients without AL who were not treated with adjuvant chemotherapy (RER: 1.85, 95% CI: 1.36–2.51, Table 3). Stage III CC patients with AL who were treated with adjuvant chemotherapy had a higher RER for death compared with CC patients without AL who were treated with adjuvant chemotherapy (RER: 1.37, 95% CI: 1.06–1.77, Table 3). Median length of follow-up for CC patients was 4.2 years (IQR: 2.3–6.8 y).

**TABLE 2:** Multivariable Relative Excess Risk (RER) for Death After Colorectal Resection

	Colon Cancer			Rectum Cancer		
	RER	95% CI	*P*	RER	95% CI	*P*
Anastomotic leakage						
No anastomotic leakage	**1 (ref.)**			**1 (ref.)**		
Anastomotic leakage	1.22	1.01–1.34	**<0.01**	1.56	1.34–1.81	**<0.01**
Gender						
Male	**1 (ref.)**			**1 (ref.)**		
Female	1.06	1.01–1.11	**0.03**	0.97	0.88–1.07	0.58
Age						
<70 y	**1 (ref.)**			**1 (ref.)**		
≥70 y	1.17	1.11–1.23	**<0.01**	1.27	1.15–1.41	**<0.01**
Surgical approach						
Open	**1 (ref.)**			**1 (ref.)**		
Laparoscopic	0.68	0.64–0.71	**<0.01**	0.65	0.59–0.72	**<0.01**
Robot-assisted	0.37	0.11–1.22	0.10	0.23	0.04–1.44	0.12
Pathological tumor stage						
Stage 1	**1 (ref.)**			**1 (ref.)**		
Stage 2	4.11	2.74–6.18	**<0.01**	10.86	2.27–51.93	**<0.01**
Stage 3	19.39	12.99–28.94	**<0.01**	38.10	8.04–180.45	**<0.01**
Stage 4	93.8	62.92–139.99	**<0.01**	172.91	36.48–819.50	**<0.01**
Type of resection						
Ileocecal resection/right hemicolectomy	**1 (ref.)**					
Transversectomy	0.84	0.72–0.99	**0.04**			
(Extended) left hemicolectomy	0.67	0.57–0.79	**<0.01**			
Sigmoid resection	0.69	0.65–0.73	**<0.01**			
Subtotal resection	1.13	0.99–1.30	0.07			
Neoadjuvant radiotherapy						
No neoadjuvant radiotherapy				**1 (ref.)**		
Neoadjuvant radiotherapy				0.74	0.65–0.84	**<0.01**
Neoadjuvant chemoradiation						
No neoadjuvant chemoradiation				**1 (ref.)**		
Neoadjuvant chemoradiation				0.98	0.86–1.12	0.74
Adjuvant chemotherapy						
No adjuvant chemotherapy	**1 (ref.)**					
Adjuvant chemotherapy	0.80	0.76–0.84	**<0.01**			

Bold values indicate a significance level of *P*<0.05.

Analyses were performed for colon cancer patients and rectal cancer patients separately.

**TABLE 3:** Multivariable Relative Excess Risk (RER) for Death After Anastomotic Leakage Stratified Per Tumor Stage

	Colon			Rectum		
Anastomotic leakage	RER	95% CI	*P*	RER	95% CI	*P*
Stage 1	1.90	0.72–5.02	0.19	3.37	0.53–21.25	0.20
Stage 2	1.83	1.37–2.43	**<0.01**	1.71	0.97–3.00	0.06
Stage 2 with adjuvant chemotherapy	1.29	0.52–3.22	0.58			
Stage 2 without adjuvant chemotherapy	1.85	1.36–2.51	**<0.01**			
Stage 3	1.27	1.09–1.48	**<0.01**	1.61	1.32–1.96	**<0.01**
Stage 3 with adjuvant chemotherapy	1.37	1.06–1.77	**0.02**			
Stage 3 without adjuvant chemotherapy	1.19	0.98–1.44	0.08			
Stage 4	0.96	0.83–1.12	0.62	1.36	1.04–1.77	**0.02**

Bold values indicate statistically significant *P*>0.005.

No anastomotic leak=reference category

Five-year relative survival for RC patients with and without AL was 97% versus 101% for stage I (*P*=1.00), 90% versus 95% for stage II (HR: 1.51, 95% CI: 0.84–2.70, *P*=0.17), 74% versus 83% for stage III (HR: 1.53, 95% CI: 1.27–1.86, *P*<0.01), and 32% versus 41% for stage IV (HR: 1.27, 95% CI: 0.99–1.63, *P*=0.06, Fig. 1). Multivariable RER for death after RC resection was significantly higher for patients with AL (Table 2). Compared with patients without AL with the same pathological tumor stage, AL patients with stage III and IV RC had a higher RER for death (RER: 1.61, 95% CI: 1.32–1.96 and RER: 1.36, 95% CI: 1.04–1.77, Table 3). Median length of follow-up for RC patients was 5.0 years (IQR: 3.0–7.7 y).

### DFS and Disease Recurrence

In 2015, 10,139 CRC patients underwent a resection with formation of a primary anastomosis, of whom 5387 were operated in the first semester of 2015. After excluding stage IV patients (1036), and patients who died within 90 days (102), a total of 4249 patients remained (Supplementary Fig. 1, Supplemental Digital Content 2, http://links.lww.com/SLA/E116).

In total, 3297 CC stage I to III patients were analyzed, including 151 patients with AL. Four-year DFS was significantly lower for CC patients with AL (79.2%) compared with patients without AL (84.7%, *P*=0.04, Supplementary Fig. 2, Supplemental Digital Content 3, http://links.lww.com/SLA/E117). Multivariable cox proportional hazard regression demonstrated that AL was not associated with disease recurrence (HR: 1.36, 95% CI: 0.94–1.97, *P*=0.10, Table 4).

**TABLE 4:** Univariable and Multivariable Cox Proportional Hazard Regression to Assess the Association Between AL and Disease Recurrence After Colon Cancer and Rectal Cancer Resection

	Colon							Rectum					
	Univariable				Multivariable			Univariable			Multivariable		
		Hazard Ratio	95% CI	*P*	Hazard Ratio	95% CI	*P*	Hazard Ratio	95% CI	*P*	Hazard Ratio	95% CI	*P*
Anastomotic leakage	No	**1 (ref.)**			**1 (ref.)**			**1 (ref.)**			**1 (ref.)**		
	Anastomotic leakage	1.28	0.95–1.74	0.11	1.36	0.94–1.97	0.10	0.95	0.55–1.65	0.87	0.91	0.53–1.57	0.73
Gender	Male	**1 (ref.)**						**1 (ref.)**					
	Female	0.91	0.77–1.09	0.32				1.01	0.75–1.37	0.93			
Age	<70 y	**1 (ref.)**			**1 (ref.)**			**1 (ref.)**					
	≥70 y	1.22	1.02–1.45	0.03	1.19	0.99–1.41	0.06	1.17	0.87–1.59	0.30			
Pathological tumor stage	1	**1 (ref.)**			**1 (ref.)**			**1 (ref.)**			**1 (ref.)**		
	2	4.09	2.75–6.09	**<0.01**	3.80	2.55–5.67	**<0.01**	2.39	1.31–4.36	**<0.01**	2.74	1.48–5.08	**<0.01**
	3	9.81	6.71–14.32	**<0.01**	9.11	6.22–13.34	**<0.01**	4.87	2.90–8.18	**<0.01**	5.90	3.40–10.24	**<0.01**
Tumor differentiation	Well differentiated	**1 (ref.)**			**1 (ref.)**			**1 (ref.)**					
	Moderately differentiated	1.31	0.72–2.38	0.38	1.03	0.59–1.97	0.93	0.72	0.32–1.62	0.42			
	Poor differentiated	3.06	1.63–5.73	**<0.01**	1.89	1.01–3.60	**<0.05**	1.20	0.46–3.15	0.72			
	Anaplastic	3.38	0.44–26.21	0.24	1.75	0.23–13.58	0.59	0.63	0.25–1.57	0.32			
Neoadjuvant radiotherapy	No							**1 (ref.)**			**1 (ref.)**		
	Yes							1.35	1.01–1.81	**<0.05**	0.74	0.54–1.01	0.06
Neoadjuvant chemoradiation	No							**1 (ref.)**					
	Yes							1	0.96–1.76	0.10			

Bold values indicate statistically significant *P*>0.005.

In total, 952 RC stage I to III patients were analyzed, including 76 patients with AL. Four-year DFS was 81.4% for RC patients with AL and 80.2% for patients without AL (*P*=0.87, Supplementary Fig. 2, Supplemental Digital Content 3, http://links.lww.com/SLA/E117). Multivariable cox proportional hazard regression revealed that AL was not associated with disease recurrence (HR: 0.91, 95% CI: 0.53–1.57, *P*=0.73, Table 4).

## DISCUSSION

In this largest population-based study published so far, AL was associated with a reduced survival in stage II and III CC patients and stage III and IV RC patients. In a subset of CRC patients, DFS was significantly decreased in CC patients with AL, but no association was found between AL and disease recurrence during 4-year follow-up.

Evidence is scarcely available on the association between AL and relative survival after CRC resection. Contrary to overall survival, relative survival can be useful to evaluate the effect of AL on survival because it adjusts for general life expectancy and is an approximation of cancer-specific survival However, only a few studies reported relative or cancer-specific survival rates and could be affected by death due to other causes.^12^,^21^ Although the evidence is scarce, a meta-analysis by Mirnezami et al^13^ demonstrated in 4 out of 6 included studies (5329 patients) a significantly reduced disease-specific survival following AL after RC resection (OR ranging 1.10–2.23).

The pathophysiological mechanisms behind the association between AL and decreased survival in stage III RC patients remains speculative. To increase resectability and decrease local recurrences these patients undergo neoadjuvant (chemo)radiotherapy. However, neoadjuvant (chemo)radiotherapy itself is associated with AL.^22^,^23^ Theoretically, a combination of neoadjuvant therapy, surgical resection, and subsequent AL might have a detrimental effect on the postoperative immune response and thereby delaying recovery and compromising general health.

CC patients with AL and treated with adjuvant chemotherapy had a significantly worse survival compared with patients without AL. Stormark et al^12^ showed similar results after analyzing the association between AL and 5-year relative survival in >22,000 stage I to III CC patients. Survival benefit of adjuvant chemotherapy in CC patients seems to be highest when started within 6 to 8 weeks after resection.^24^,^25^ Adjuvant chemotherapy can lead to a reduction of disease recurrence up to 50% compared with patients who solely underwent surgery and is recommended for high risk stage II and III disease.^26^ Since AL develops in the postoperative phase it could have postponed the initiation or led to cancellation of adjuvant chemotherapy, and thereby reducing survival. This may also explain our finding that pathological stages II and III were associated with an increased risk of disease recurrence.

Surgical resection for stage IV CRC can be performed as intentional curative treatment in combination with local treatment of metastases, or to prevent or treat tumor complications. Previous studies demonstrated that palliative resection of the primary tumor can improve overall survival in stage IV CRC patients.^27^ However, this improvement was not demonstrated in recent RCTs.^28^ In accordance with previous studies, this study showed that survival worsens if AL occurs in stage IV RC patients. Clinical deterioration and surgical trauma-induced immunosuppression as a result of AL may induce disease progression in patients with metastatic disease.^29^ This might be an argument to be reluctant performing palliative surgery of the primary tumor in stage IV patients.^30^ Although not in line with previous studies,^31^,^32^ this study showed an association between open surgery and worse oncological outcomes. Traditionally, CRC patients are operated using (robot-assisted) laparoscopy in the Netherlands, whereas open surgery is only performed in case of advanced tumor stages or in an emergency setting. In the present study, stage IV CRC patients underwent significantly more open and emergency resections (data not shown). Advanced tumor stages and emergency resections are independently associated with a higher risk of developing AL.^10^,^33^–^37^ Therefore, it is reasonable to assume that in case of open (emergency) resections, advanced tumor stages and development of AL confounded the results.

DFS was significantly decreased in CC patients with AL, however, no association between AL and disease recurrence was found. Previous smaller cohort studies reported contradictory results on disease recurrence after CRC resection.^15^–^17^ Their main finding was that AL was associated with distant recurrence in CC patients and local recurrence in RC patients, which was substantiated by the theory that AL promotes viable tumor cells to retain their oncological competence by immunosuppression.^3^,^17^,^21^ These contradictory results can be explained by oncological outcomes being influenced by characteristics such as poor tumor differentiation and higher pathological stages,^10^ which is confirmed by the present study.

This study has strengths and limitations. A large number of patients who underwent CRC surgery with a primary anastomosis were included and separate analyses for both entities were performed. However, AL was only registered if a reintervention or readmission was required within 60 days after primary surgery and a considerable number of patients develop AL thereafter. This phenomenon is mainly observed in patients who received a diverting ileostomy, which is known to diminish the severity of AL.^1^ Lately diagnosed ALs can either heal with conservative management, or might develop into a chronic presacral sinus requiring salvage surgery. Not including those late leaks in our study might have affected long-term outcomes to some extent. DFS and disease recurrence were analyzed in a relatively smaller cohort. The relatively low rates of disease recurrence might have led to insufficient statistical power to detect significant differences. These results should be interpreted with caution, albeit several other studies also failed to show a significant impact of AL on disease recurrence.

In conclusion, AL was associated with a negative impact on survival in stage II and III CC patients, and in stage III and IV RC patients. DFS was significantly decreased in CC patients with AL, but no association was found between AL and disease recurrence in CRC patients. To mitigate the negative impact of AL on long-term outcomes after CRC surgery, nonrestorative surgery can be considered in patients at high risk of AL. Further studies have to elucidate the pathophysiological mechanism of AL, to develop early detection techniques and to investigate treatment strategies to reduce the impact of AL on oncological outcomes.

## Supplementary Material

SUPPLEMENTARY MATERIAL

## DISCUSSANTS

### Ronan P. O’Connell (Dublin, Ireland)

The authors are to be congratulated for the mammoth task of analysis undertaken; However, the value of the findings, in the context of providing a benchmark for future studies, requires careful scrutiny.

The authors have elected to define anastomotic leakage (AL) as leakage of abdominal content or abscess formation at the site of the anastomosis requiring reoperation, radiological intervention or readmission within 60 days after anastomosis formation. This clearly excludes patients with AL, who did not require radiological or surgical reintervention. They have also excluded patients who died within 90 days of surgery. Thus, the seemingly very low rates of AL presented (4.8% and 7.9% for colonic and rectal anastomosis, respectively) must be seen in the context of significant exclusion criteria that undermine the purpose of the study. Could the authors kindly comment on how their findings might reduce the incidence and impact of AL?

The concept of relative survival is important and infrequently employed in the analysis of cancer outcomes. It is intuitive that patients with more advanced disease, in whom adjuvant therapies were delayed due to complications, might not have the same relative survival as those whose adjuvant therapy was not delayed; however, it is helpful to know that, in those with stage 1 disease, there was no adverse outcome. Of course, this, too, is intuitive given the expected high cure rate of resection of stage 1 disease.

It is not clear why open surgical resection was associated with worse oncological outcomes, unless there is an inherent bias in case selection that is not accounted for. Equally, it is not clear why age under 70 had worse outcomes, unless, again, there is a selection bias, in that older frailer patients are more likely to have a non-restorative resection. Could the authors respond to these points?

### Response from Nynke Greijdanus (Nijmegen, The Netherlands)

Thank you for your remarks and questions. First, regarding relative survival, we believe that this is a better measure compared to overall survival, which is more frequently used in other papers. We adjusted for the mortality in the general population, and by doing so, the rate of mortality is more cancer-specific compared to overall survival. Of course, overall survival takes all deaths into account, but you cannot really pinpoint whether this was due to disease recurrence or to the disease itself. So, by using relative survival, we hope to give a better cancer-specific representation.

Second, regarding emergency resections, we agree that this can be influenced by other factors. In the Netherlands, patients with colorectal cancer are traditionally treated by laparoscopic surgery or robot-assisted surgery. In the case of open resections, this is predominantly performed for emergency resections or for patients with advanced disease. Our own data confirm that the patients who underwent an emergency resection were mostly treated with an open resection, as were most patients with stage 4 disease.

Regarding younger patients, we saw that age below 70 was associated with a higher risk of developing anastomotic leakage. We think that this can be selection bias because elderly patients might be frailer or have more comorbidities; therefore, surgeons might opt for non-restorative surgery in this cohort.

### Nicolò de Manzini (Trieste, Italy)

Please receive all my compliments for the huge work you have done with such an important series. In effect, it seems strange that the leakage doesn’t affect the recurrence. However, based on previous studies, we know that any complication that delays chemotherapy by more than 90 days in Stage 3 colon cancer significantly influences overall survival. Would you, perhaps, be able to differentiate between leakages with a rapid healing and those with strong complications that delay chemotherapy?

### Response from Nynke Greijdanus (Nijmegen, The Netherlands)

Thank you for your question. We can’t differentiate between healing time because we did not look at this. However, we saw that the patients, who had an anastomotic leakage and received chemotherapy, had a worse survival rate. So, we believe that anastomotic leakage is caused by several risk factors, such as smoking, medications used, and other comorbidities, and that these factors are also associated with disease recurrence and tumor biology. In other words, we think that anastomotic leakage patients are patients who have a poorer general health overall, and are, therefore, more prone to develop disease recurrence.

### Georgios Sotiropoulos (Athens, Greece)

Thank you very much for your nice presentation. I have a clinical question. In your study, did you include Stage 4 patients with simultaneous liver resection using the Pringle maneuver, which could also affect anastomotic leakage?

### Response from Nynke Greijdanus (Nijmegen, The Netherlands)

That’s an interesting question. However, we did not look at this.

## References

[1] Borstlap WAA Westerduin E Aukema TS. Dutch Snapshot Research G. Anastomotic leakage and chronic presacral sinus formation after low anterior resection: results from a large cross-sectional study. Ann Surg. 2017;266:870–877. 28746154 10.1097/SLA.0000000000002429

[2] Krarup PM Jorgensen LN Andreasen AH. A nationwide study on anastomotic leakage after colonic cancer surgery. Colorectal Dis. 2012;14:e661–e667. 22564292 10.1111/j.1463-1318.2012.03079.x

[3] Krarup PM Nordholm-Carstensen A Jorgensen LN. Anastomotic leak increases distant recurrence and long-term mortality after curative resection for colonic cancer: a nationwide cohort study. Ann Surg. 2014;259:930–938. 24045445 10.1097/SLA.0b013e3182a6f2fc

[4] Benlice C Gorgun E Liu X. Risk factors of anastomotic leak after colectomy: a nationwide study. J Am Coll Surg. 2014;219:e76–e77.

[5] Cong Z-J Hu L-H Bian Z-Q. Systematic review of anastomotic leakage rate according to an international grading system following anterior resection for rectal cancer. PLoS One. 2013;8:e75519. 24086552 10.1371/journal.pone.0075519 PMC3783382

[6] Frasson M Granero-Castro P Ramos Rodríguez JL. Risk factors for anastomotic leak and postoperative morbidity and mortality after elective right colectomy for cancer: results from a prospective, multicentric study of 1102 patients. Int J Colorectal Dis. 2016;31:105–114. 26315015 10.1007/s00384-015-2376-6

[7] Turrentine FE Denlinger CE Simpson VB. Morbidity, mortality, cost, and survival estimates of gastrointestinal anastomotic leaks. J Am Coll Surg. 2015;220:195–206. 25592468 10.1016/j.jamcollsurg.2014.11.002

[8] Arron MNN Greijdanus NG Ten Broek RPG. Trends in risk factors of anastomotic leakage after colorectal cancer surgery (2011-2019): a Dutch population-based study. Colorectal Dis. 2021;23:3251–3261. 34536987 10.1111/codi.15911 PMC9293104

[9] Ramphal W Boeding JRE Gobardhan PD. Oncologic outcome and recurrence rate following anastomotic leakage after curative resection for colorectal cancer. Surg Oncol. 2018;27:730–736. 30449500 10.1016/j.suronc.2018.10.003

[10] Ebinger SM Warschkow R Tarantino I. Anastomotic leakage after curative rectal cancer resection has no impact on long-term survival: a propensity score analysis. Int J Colorectal Dis. 2015;30:1667–1675. 26245949 10.1007/s00384-015-2331-6

[11] Crippa J Duchalais E Machairas N. Long-term oncological outcomes following anastomotic leak in rectal cancer surgery. Dis Colon Rectum. 2020;63:769–777. 32109914 10.1097/DCR.0000000000001634

[12] Stormark K Krarup PM Sjövall A. Anastomotic leak after surgery for colon cancer and effect on long-term survival. Colorect Dis. 2020;22:1108–1118. 10.1111/codi.14999 32012414

[13] Mirnezami A Mirnezami R Chandrakumaran K. Increased local recurrence and reduced survival from colorectal cancer following anastomotic leak: systematic review and meta-analysis. Ann Surg. 2011;253:890–899. 21394013 10.1097/SLA.0b013e3182128929

[14] Boccola MA Buettner PG Rozen WM. Risk factors and outcomes for anastomotic leakage in colorectal surgery: a single-institution analysis of 1576 patients. World J Surg. 2011;35:186–195. 20972678 10.1007/s00268-010-0831-7

[15] Koedam TWA Bootsma BT Deijen CL. Oncological outcomes after anastomotic leakage after surgery for colon or rectal cancer: increased risk of local recurrence. Ann Surg. 2022;275:e420–e427. 32224742 10.1097/SLA.0000000000003889

[16] den Dulk M Marijnen CA Collette L. Multicentre analysis of oncological and survival outcomes following anastomotic leakage after rectal cancer surgery. Br J Surg. 2009;96:1066–1075. 19672927 10.1002/bjs.6694

[17] Hain E Maggiori L Manceau G. Oncological impact of anastomotic leakage after laparoscopic mesorectal excision. Br J Surg. 2017;104:288–295. 27762432 10.1002/bjs.10332

[18] Rahbari NN Weitz J Hohenberger W. Definition and grading of anastomotic leakage following anterior resection of the rectum: a proposal by the International Study Group of Rectal Cancer. Surgery. 2010;147:339–351. 20004450 10.1016/j.surg.2009.10.012

[19] Seppä K Hakulinen T Läärä E. Comparing net survival estimators of cancer patients. Stat Med. 2016;35:1866–1879. 26707551 10.1002/sim.6833

[20] Ederer F Heise H. Instructions to IBM 650 Programmers in Processing Survival Computations Methodological Note No 10, End Results Evaluation Section. Bethesda, MD: National Cancer Institute; 1959.

[21] Hüttner FJ Warschkow R Schmied BM. Prognostic impact of anastomotic leakage after elective colon resection for cancer—a propensity score matched analysis of 628 patients. Eur J Surg Oncol. 2018;44:456–462. 29396327 10.1016/j.ejso.2018.01.079

[22] Matthiessen P Hallbook O Andersson M. Risk factors for anastomotic leakage after anterior resection of the rectum. Colorectal Dis. 2004;6:462–469. 15521937 10.1111/j.1463-1318.2004.00657.x

[23] Zaborowski AM Stakelum A Winter DC. Anastomotic leak risk in complete responders to neoadjuvant therapy for rectal cancer: a systematic review. Int J Colorectal Dis. 2021;36:671–676. 33427960 10.1007/s00384-021-03833-w

[24] Turner MC Farrow NE Rhodin KE. Delay in adjuvant chemotherapy and survival advantage in stage III colon cancer. J Am Coll Surg. 2018;226:670–678. 29378259 10.1016/j.jamcollsurg.2017.12.048

[25] Gao P Huang XZ Song YX. Impact of timing of adjuvant chemotherapy on survival in stage III colon cancer: a population-based study. BMC Cancer. 2018;18:234. 29490625 10.1186/s12885-018-4138-7 PMC5831576

[26] Lima IS Yasui Y Scarfe A. Association between receipt and timing of adjuvant chemotherapy and survival for patients with stage III colon cancer in Alberta, Canada. Cancer-Am Cancer Soc. 2011;117:3833–3840. 10.1002/cncr.25954 21319156

[27] Lam-Boer J Van der Geest LG Verhoef C. Palliative resection of the primary tumor is associated with improved overall survival in incurable stage IV colorectal cancer: a nationwide population-based propensity-score adjusted study in the Netherlands. Int J Cancer. 2016;139:2082–2094. 27342618 10.1002/ijc.30240

[28] van der Kruijssen DEW Elias SG Vink GR. Sixty-day mortality of patients with metastatic colorectal cancer randomized to systemic treatment vs primary tumor resection followed by systemic treatment: The CAIRO4 Phase 3 Randomized Clinical Trial. JAMA Surg. 2021;156:1093–1101. 34613339 10.1001/jamasurg.2021.4992 PMC8495602

[29] Smith JD Butte JM Weiser MR. Anastomotic leak following low anterior resection in stage IV rectal cancer is associated with poor survival. Ann Surg Oncol. 2013;20:2641–2646. 23385965 10.1245/s10434-012-2854-9

[30] Fong YM. Primary tumor resection and patients with asymptomatic colorectal cancer and nonresectable metastases results of recent randomized trials. JAMA Surg. 2021;156:1101–1102. 34613343 10.1001/jamasurg.2021.5023

[31] Deijen CL Vasmel JE de Lange-de Klerk ESM. Ten-year outcomes of a randomised trial of laparoscopic versus open surgery for colon cancer. Surg Endosc. 2017;31:2607–2615. 27734203 10.1007/s00464-016-5270-6 PMC5443846

[32] Bonjer HJ Deijen CL Haglind E. Group CIS. A randomized trial of laparoscopic versus open surgery for rectal cancer. N Engl J Med. 2015;373:194. 10.1056/NEJMc1505367 26154803

[33] McDermott FD Heeney A Kelly ME. Systematic review of preoperative, intraoperative and postoperative risk factors for colorectal anastomotic leaks. Br J Surg. 2015;102:462–479. 25703524 10.1002/bjs.9697

[34] Lee CHA Kong JCH Heriot AG. Short-term outcome of emergency colorectal cancer surgery: results from Bi-National Colorectal Cancer Audit. Int J Colorectal Dis. 2019;34:63–69. 30269226 10.1007/s00384-018-3169-5

[35] Baer C Menon R Bastawrous S. Emergency presentations of colorectal cancer. Surg Clin North Am. 2017;97:529–545. 28501245 10.1016/j.suc.2017.01.004

[36] Bass G Fleming C Conneely J. Emergency first presentation of colorectal cancer predicts significantly poorer outcomes: a review of 356 consecutive Irish patients. Dis Colon Rectum. 2009;52:678–684. 19404074 10.1007/DCR.0b013e3181a1d8c9

[37] Chiarugi M Galatioto C Panicucci S. Oncologic colon cancer resection in emergency: are we doing enough? Surg Oncol. 2007;16(suppl 1 S). 73–S77. 10.1016/j.suronc.2007.10.019 18032028

