# Supplementary figures and images for: Long-Term Oncological Outcomes After Colorectal Anastomotic Leakage: A Retrospective Dutch Population-based Study
Source: Ann Surg. 2022 Aug 5;276(5):882–9. doi: 10.1097/SLA.0000000000005647 (PMC9534056; doi:10.1097/SLA.0000000000005647)

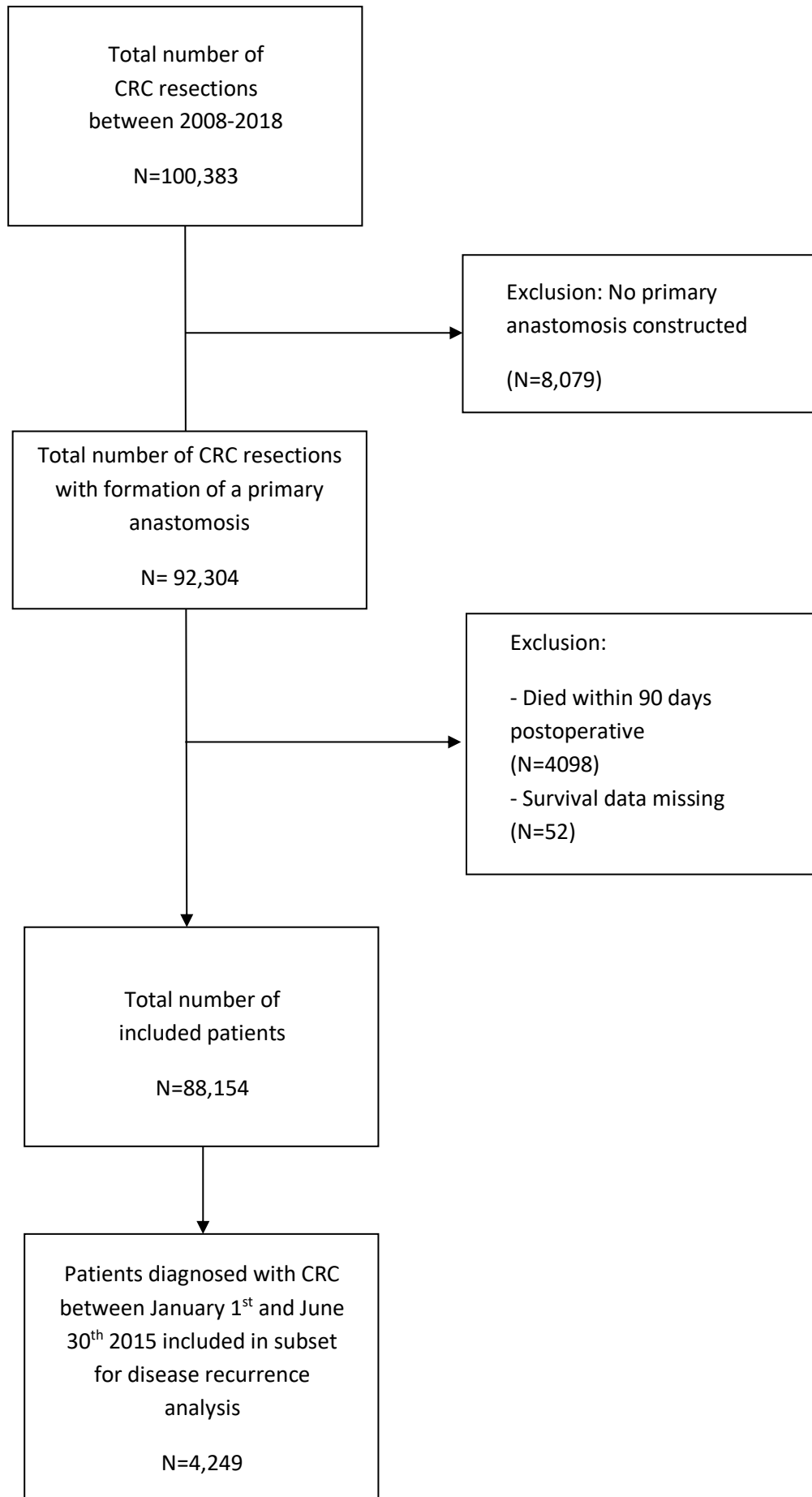

Supplement: SUPPLEMENTARY MATERIAL [file sla-276-0882-s001.pdf]

## Colon cancer

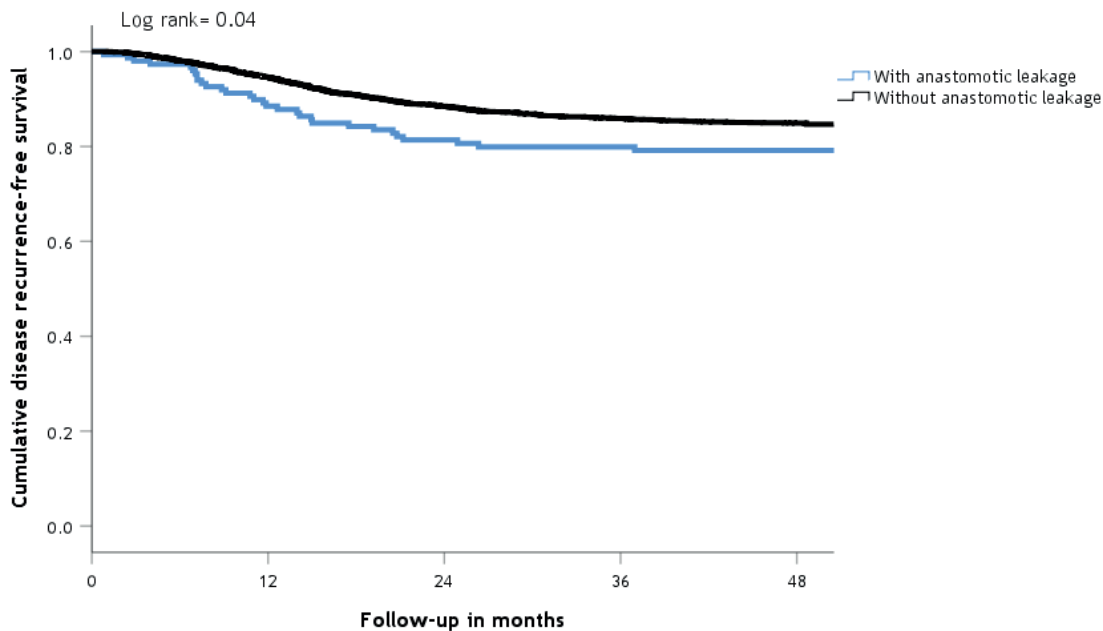

## Rectal cancer

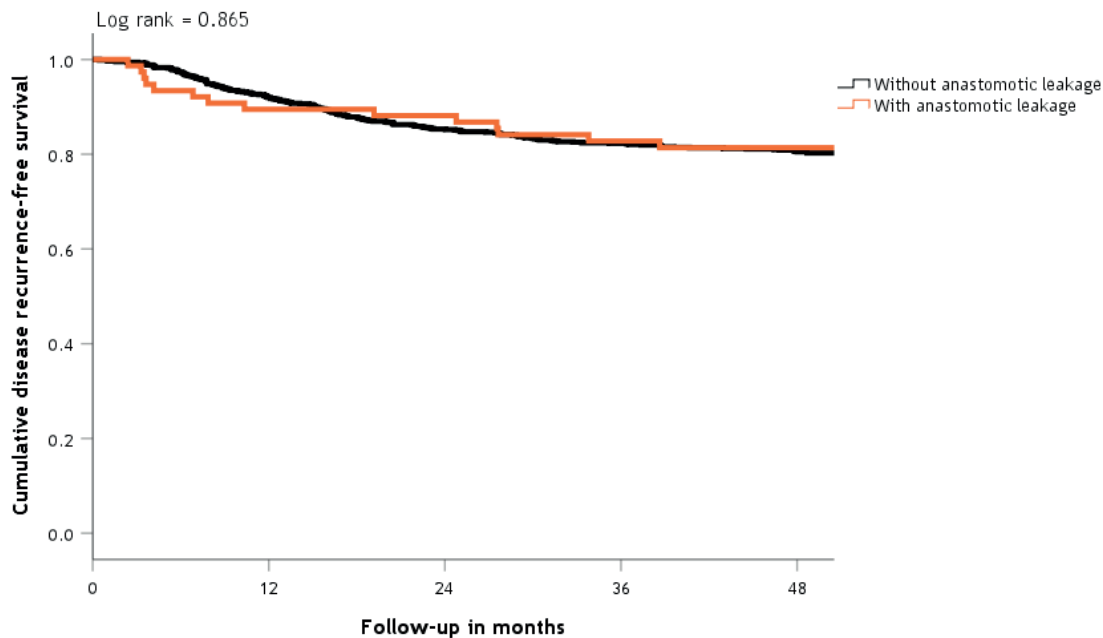

Supplement: SUPPLEMENTARY MATERIAL [file sla-276-0882-s002.pdf]
